# Providing Self-Healing Ability for Wireless Sensor Node by Using Reconfigurable Hardware

**DOI:** 10.3390/s121114570

**Published:** 2012-10-29

**Authors:** Shenfang Yuan, Lei Qiu, Shang Gao, Yao Tong, Weiwei Yang

**Affiliations:** The State Key Lab of Mechanics and Control of Mechanical Structures, Nanjing University of Aeronautics and Astronautics, 29# Yu Dao Street, Nanjing 210016, China; E-Mails: ql19830925@nuaa.edu.cn (L.Q.); gsnuaa2003@163.com (S.G.); tongyao@nuaa.edu.cn (Y.T.); yww222@nuaa.edu.cn (W.Y.)

**Keywords:** wireless sensor node, self-healing, reconfigurable hardware, dynamical reconfiguration

## Abstract

Wireless sensor networks (WSNs) have received tremendous attention over the past ten years. In engineering applications of WSNs, a number of sensor nodes are usually spread across some specific geographical area. Some of these nodes have to work in harsh environments. Dependability of the Wireless Sensor Network (WSN) is very important for its successful applications in the engineering area. In ordinary research, when a node has a failure, it is usually discarded and the network is reorganized to ensure the normal operation of the WSN. Using appropriate WSN re-organization methods, though the sensor networks can be reorganized, this causes additional maintenance costs and sometimes still decreases the function of the networks. In those situations where the sensor networks cannot be reorganized, the performance of the whole WSN will surely be degraded. In order to ensure the reliable and low cost operation of WSNs, a method to develop a wireless sensor node with self-healing ability based on reconfigurable hardware is proposed in this paper. Two self-healing WSN node realization paradigms based on reconfigurable hardware are presented, including a redundancy-based self-healing paradigm and a whole FPAA/FPGA based self-healing paradigm. The nodes designed with the self-healing ability can dynamically change their node configurations to repair the nodes' hardware failures. To demonstrate these two paradigms, a strain sensor node is adopted as an illustration to show the concepts. Two strain WSN sensor nodes with self-healing ability are developed respectively according to the proposed self-healing paradigms. Evaluation experiments on self-healing ability and power consumption are performed. Experimental results show that the developed nodes can self-diagnose the failures and recover to a normal state automatically. The research presented can improve the robustness of WSNs and reduce the maintenance cost of WSNs in engineering applications.

## 1. Introduction

Wireless sensor networks (WSNs) have received tremendous attention over the past ten years [1–6]. The recent developments in micro-electromechanical-systems have led to the rapid production of many inexpensive sensors. Sensor nodes are typically equipped with on-board processors which offer distributed processing and computational ability. A WSN usually consists of a large number of spatially distributed autonomous sensor nodes used to cooperatively monitor physical or environmental conditions, such as temperature, sound, vibration, pressure, motion and pollutants. WSN technology has found very important applications both in military and civilian application areas such as battlefield surveillance, industrial process monitoring and control, structural health monitoring, environment and habitat monitoring, healthcare applications, home automation, and environmental control in buildings [7].

In engineering applications, a number of sensor nodes are usually spread across a geographical area of interest. Some of these nodes have to work in harsh environments and for a long term. For example, in the civil structural health monitoring area, large civil engineering infrastructures, such as bridges, highways, tunnels and water pipes, are expected to last for decades or even centuries. Over the course of their lifetimes, these structures deteriorate and require timely maintenance in order to prevent further degradation that might lead to accidents, the need for replacement or, in the worst case, collapse. Traditionally, early detection of such deterioration is achieved by visual inspection, either during routine maintenance visits or when a maintenance team is sent to the site to investigate a known or suspected problem. However, such inspections are time-consuming and costly. An alternative is to equip infrastructure with sensors that are permanently wired up to report back to a central system. But this solution is not adopted very extensively because of the difficulty and cost of running data and power cables to each individual sensor in challenging environments such as a subway tunnel or a long suspension bridge. Hence, WSN based monitoring systems have obvious advantages in this application area and the WSN sensor nodes are usually placed in a distributed manner on different sites on these large scale structures, working in a natural environment and are usually expected to be in service during the whole life time of these structures. Robustness of the WSN itself is then critical.

Regarding the robustness of WSNs, plenty of research has been reported concentrating on network re-organization methods when one or several nodes fail [8–12]. The authors of [10] propose a framework for an evolvable sensor network architecture, investigated as part of the ESPACENET project, collocated at the University of Edinburgh, Essex, Kent and Surrey, UK. A policy controlled self-configuration method in unattended WSNs is presented in [11]. A lifetime extension method based intelligent redeployment method for surveillance WSNs is reported in [12]. There are also a number of topology management protocols which function on criteria such as the formation of a neighbor list, discovery of neighbor nodes and controlling the duty cycle of the sensor nodes. Using the WSN re-organization methods, though the sensor networks can be reorganized, this causes additional maintenance costa and sometimes the function of the networks still decreases. Under those situations that the sensor networks cannot be reorganized, the performance of the whole WSN will surely be degraded.

In this paper, to improve the robustness and reliability of the wireless sensor network nodes to last their service life when they have to work in a natural environment or for a long term, a hardware self-healing method is proposed to recover the WSN sensor nodes when only some parts of the nodes' hardware have faults. This method is based on reconfigurable hardware, such as Field Programmable Analog Arrays (FPAAs) and Field Programmable Gate Arrays (FPGAs), whose architecture can be dynamically reconfigured. Using the presented method, when part of the circuits in the sensor node fail, it is not necessary to abandon the whole node. The node can diagnose its own fault and repair itself. This can greatly improve the robustness of WSNs when nodes are working in a geographically large and harsh area and reduce the maintenance costs of the WSNs.

The structure of this paper is as follows: Section 2 introduces related work. Section 3 proposes two realization paradigms for self-heading sensor nodes based on reconfigurable hardware. The design and implementation of two self-healing WSN strain sensor nodes based on the redundancy hardware self-healing paradigm and the whole reconfigurable hardware based paradigm are explained in detail in Section 4. Section 5 introduces the experimental demonstration of the failure diagnostic process and the self-healing results. The power consumption of the developed self-healing nodes is also measured and discussed in this section. Section 6 gives the conclusions of the paper.

## 2. Related Work

Some literatures have reported the concept of self-healing WSNs [13–15]. These researches have shown that the self-healing WSN can be achieved by deploying mobile nodes [13], designing biological systems [14] and employing self-healing services [15]. However these researches still consider discarding the fault nodes, concentrating on the self-healing of the network and usually deal with the energy exhaustion problem. Under some WSN application scenarios, such as structural health monitoring, industrial process monitoring, healthcare applications, home automation, *etc.*, the batteries of wireless sensor nodes can be replaced at a certain interval or recharged. Under these situations, energy is no longer a very critical problem, but these nodes may still have to work in harsh environments or serve for a long term. The node circuits may suffer from failures during their long service life. Improving the robustness of the sensor nodes is very important to implement large scale WSNs.

FPGA for the digital domain and FPAA for the analog domain are two main kinds of reconfigurable hardware. A powerful feature of FPGAs/FPAAs is that their hardware can be reconstructed dynamically to adapt to different applications. FPGAs can be configured to implement any logical function that an ASIC could perform. FPGAs contain programmable logic components called logic blocks, and a hierarchy of reconfigurable interconnects that allow the blocks to be wired together. FPGAs provide a method for rapidly prototyping digital systems. A FPAA is an integrated device containing Configurable Analog Blocks (CAB) and interconnects between these blocks. FPAAs may be current mode or voltage mode devices. In voltage mode devices, each block usually contains an operational amplifier in combination with a programmable configuration of passive components. FPAAs provide a method for rapidly prototyping analog systems.

FPAA- or FPGA- based reconfigurable hardware systems have emerged as an important technology to improve the robustness of electric systems in recent years [16–25]. In [19] the authors present an Evolvable Hardware-based approach. The key idea is to reconfigure a programmable device, to compensate or bypass its degraded or damaged components. Goldenberg [20] reports an e-maintenance method taking advantage of the reprogrammable features of a FPGA-based reconfigurable system. A method is proposed to perform remote repair of the system by sending new firmware via Internet for reconfiguration of the FPGA. To handle the effects of single event upsets, which are common in computers in the space radiation environment, [21] introduces a new fault-tolerant system with dual-module redundancy using dynamic reconfigurable technique of FPGA. In [22] an easy way to implement reconfigurable micro-sensor interfaces for analog sensors with nonstandardized output signals based on an FPAA is introduced. Keymeulen *et al.* [23] describe a FPAA-based self-reconfigurable analog array integrated circuit architecture for space applications. In [24,25] combinations of FPAAs and FPGAs to realize both analog and digital circuit reconfigurations are presented.

Regarding the design of WSN sensor nodes, till now, plenty of literature has reported the implementation of different kinds of WSN sensor nodes [7,26–29]. Besides different kind of microprocessors, such as MSP430F149, SA1100, reconfigurable hardware, especially FPGAs, have been proposed by numerous authors to be the main processing components to design the sensor nodes [30–40]. In [30] a wireless reconfigurable smart sensor network platform for computer numerically controlled machine applications is developed. Four different smart sensors are put under test in the network and their corresponding signal processing techniques are implemented in a FPGA-based sensor node. This research takes advantages of the high processing capability of FPGAs and their reconfiguration ability to meet different specific task needs. In [31] the use of a FPGA as the processing unit to provide more powerful computing ability is suggested. Though microprocessor based wireless sensor nodes have low power consumption, they also have limited computing power which in many application cases cannot meet the needs of the complexity and number of tasks of many engineering applications. This research shows that energy saving for certain higher-end applications can be achieved. Reference [32] also mentions that the application of traditional security schemes on sensor nodes is limited due to the restricted computation capability and the inherent low data rate of ordinary microprocessor-based nodes. In order to avoid dependencies on a compromised level of security, a WSN node with a microcontroller and a FPGA is used to implement a solution based on Elliptic Curve Cryptography to improve the security. In [33], aiming at removing noisy samples during data acquisition in a WSN, a dynamically reconfigurable Kalman Filter is designed into internal Virtex-4 FPGA architecture. Other developments of FPGA-based nodes are also reported by [34–40].

The reconfigurable hardware-based WSN node design approaches described so far aim at improving the performance and flexibility of nodes. However, there is no report on realizing the hardware self-healing of WSN node by adopting the dynamic configuration ability of the reconfigurable hardware. When one component of the sensor node fails, usually the whole sensor node has to be abandoned. If the node itself can be self-healing, the robustness of the WSN could be greatly improved.

## 3. Reconfigurable Hardware Based Self-Healing WSN Node Realization Paradigms

**Figure 1.**
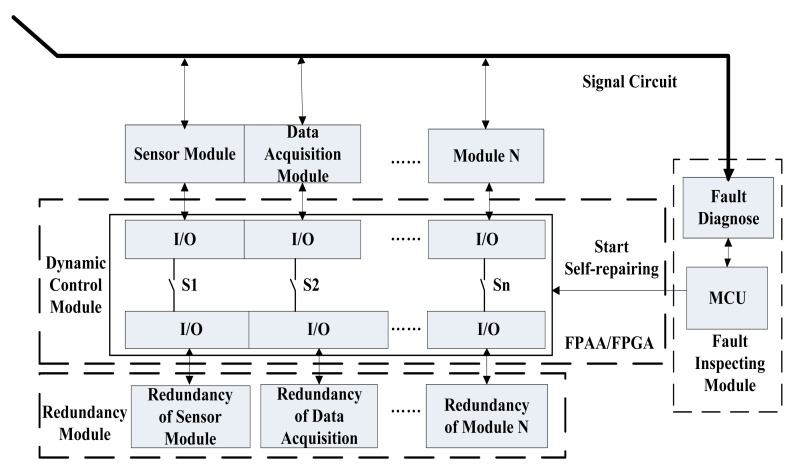
The redundancy-based self-healing WSN node paradigm.

**Figure 2.**
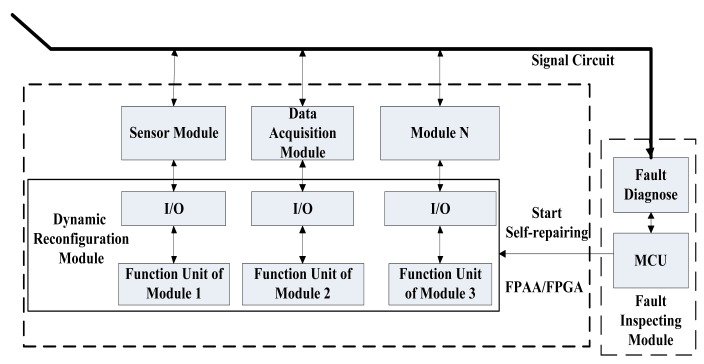
FPGA/FPAA-based self-healing WSN node design paradigm.

Since the WSN sensor node has both digital and analog circuits, FPGAs and FPAAs can be used together to realize the self-healing WSN node. Two paradigms, shown in Figures 1 and 2, are presented to realize the reconfigurable hardware-based WSN sensor nodes with self-healing ability.

Figure 1 shows a kind of redundancy-based self-healing paradigm. In this design, redundant modules of some important circuit modules are designed in the node hardware together with the FPAA or FPGA to form self-healing modules. Fault diagnostic modules are also implemented during the node design which include relevant fault diagnostic hardware and also fault diagnostic software working in the main controller on the node.

When certain part of the hardware fails, the fault diagnostic software detects the fault and the FPGA or FPAA is dynamically reconfigured to cut off the connections to the failed part and switch its redundant part into the circuits. Using this method, the defective part is abandoned and the redundant part replaces the failed module. The WSN node can be recovered to its normal status to realize a kind of self-healing ability.

Figure 2 is a whole FPGA/FPAA-based self-healing method. Using this design, the main analog and digital circuits of the WSN node are realized by the internal modules of the FPAA and FPGA, respectively. In this case, when a certain part fails, the FPGA/FPAA is dynamically reconfigured to use its other internal module to replace the failed circuit.

These two paradigms can be applied in different situations. Using the whole FPGA/FPAA-based self-healing method, no extra redundant modules are needed, which simplifies the node hardware design, and reduces the cost and dimensions of the self-healing node. However, according to the datasheets of current commercial available FPAA chips, the gain accuracy of amplifier modules in FPAA is 5%. At some high precision application scenarios, a measurement accuracy of 0.1% is required. Since the internal modules in the FPAA have limited precision, for high precision request situations, this method may not be applicable. If high node precision is needed, the redundancy-based self-healing method can be used. Since the redundancy repairing modules can have the same design as the original circuits, the precision can be ensured. The disadvantages are that the node has more complicated hardware, bigger node dimensions and more expensive cost compared to the whole FPGA/FPAA-based self-healing method.

## 4. Implementation of the Self-Healing WSN Nodes

Two WSN node self-healing paradigms have been presented in Section 3. In this section, a kind of strain WSN node is adopted to show the concept of reconfigurable hardware-based self-healing node design.

**Figure 3.**
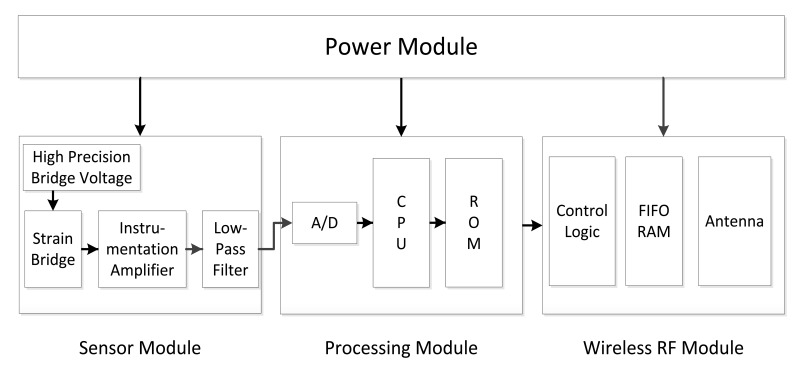
A typical WSN strain sensor node structure.

Figure 3 shows the typical WSN strain sensor node structure [5,6]. This node typically has three main modules, namely the sensor module, processing module and wireless RF module. To measure strain, strain gauges are usually adopted to form bridge circuits. The output of the bridge usually is weak and the strain signal is a low frequency signal. Hence, the sensor module of the WSN strain node usually contains three circuit parts, namely high precision voltage supply circuit, instrumentation amplifier circuit and low pass filter. The sensor module provides high stable bridge voltage to the strain bridge circuit, and also amplifies and filters the output from the bridge. The other two main modules, processing module and wireless RF module, are similar to those found in other ordinary WSN nodes. The processing module includes an A/D converter, a microprocessor and its outside circuits. This part controls the work of the whole node and also processes the data from the strain gauges. The wireless communication module takes charge of the communication with other nodes or the base station. Based on the typical strain WSN node structure, two self-healing WSN strain nodes are implemented based on the two self-healing paradigms presented in Section 1, respectively.

In the design, since amplifier and filter are two main analog circuits in the design of a lot of kinds of wireless sensor network nodes, these two hardware circuits are chosen at the design stage to simulate the hardware parts that will have failures in the later self-healing demonstration experiments as described in Table 1.

Because these two circuits are analog circuits, in the following design, a FPAA is adopted to be the reconfigurable device to perform self-healing of the node hardware circuits. In the implementation, the AN231E04FPAA chip from the Anadigm Company is chosen because of its small chip size, low energy consumption and sufficient input and output I/O.

The AN231E04, which operates with a 3.3 volt power supply with typical power in the 125 mW range, is of particular interest to this design. The AN231E04 is packaged in a 7 × 7 × 0.9 mm ultra thin 44-pin QFN (quad flat pack, no-lead) package. A key feature of the AN231E04 is that it can be dynamically reconfigured during operation by a microprocessor. The AN231E04 consists of a 2 × 2 matrix of fully configurable analog blocks, surrounded by programmable interconnect resources and analog input/output cells with active elements. Configuration data is stored in an on-chip SRAM configuration memory. Additionally, an SPI-like interface is provided for simple serial loading of configuration data from a microprocessor.

### 4.1. Sensor Node Design Using the Redundancy-Based Self-Healing Paradigm

**Figure 4.**
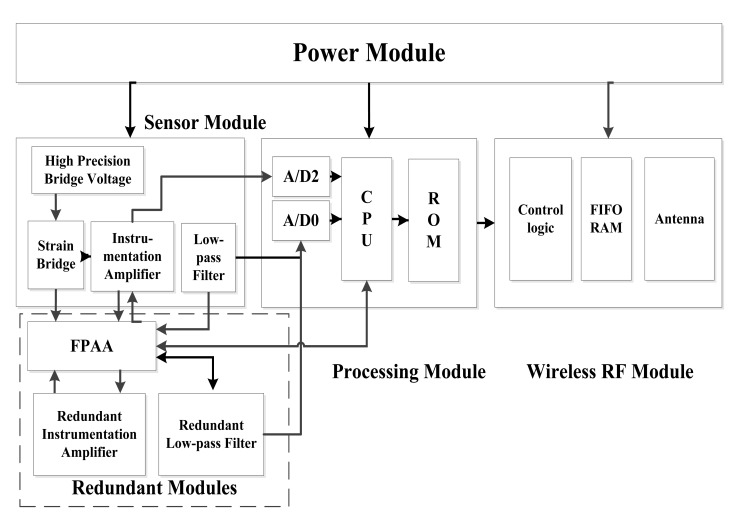
Hardware structure of the redundancy-based self-healing WSN strain node.

The schematic structure of the self-healing WSN strain sensor node designed based on the redundancy-based self-healing paradigm is shown in Figure 4. Since two analog circuits are considered here that may have self-healing needs, in the design, a redundant instrumentation amplifier and a redundant low pass filter are added to the sensor module together with the FPAA chip. In the design, all the main circuits in the sensor module are designed to be connected with the FPAA. The working of the FPAA is controlled by a microprocessor in the processing module. In order to diagnose the circuit failure, A/D0 in the microprocessor is used as the main A/D converter for converting the strain analog signal into a digital signal. Another A/D converter A/D2 is also adopted here to be connected with the output of the instrumentation amplifier for fault diagnosis.

**Figure 5.**
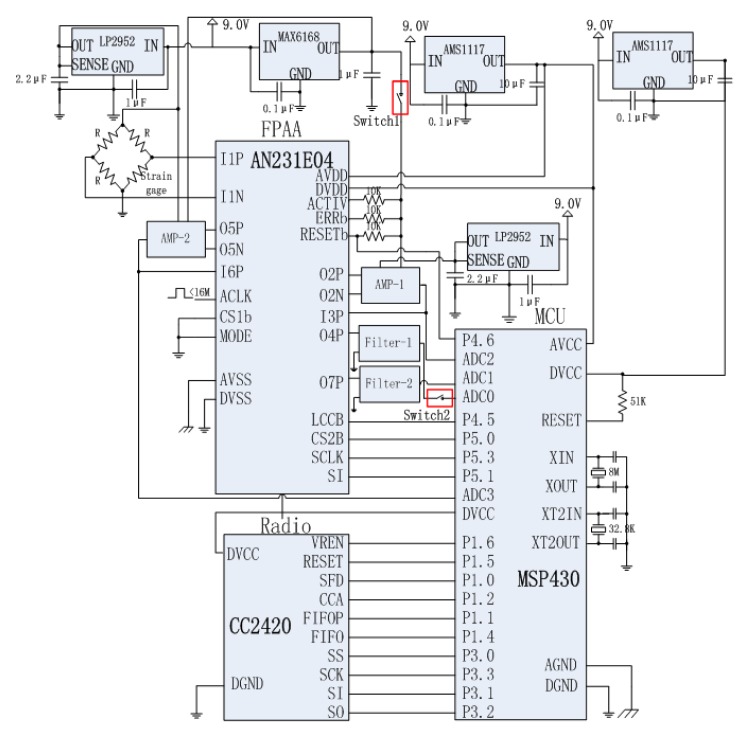
Circuit design of the redundancy-based self-healing node.

Figure 5 shows the detail circuit design of the node hardware. Four boards are designed for the self-healing WSN strain node working as sensor board, FPAA self-healing board, processing board and wireless communication board. In Figure 5, Switch 1 is used to disconnect the reference voltage input from the Ref of AD623. In this case, the AD623 saturates. Switch 2 is used to cut the connection between the output of the filter chip OPA340 and the A/D0 in the microprocessor chip.

In the design, a MSP430 microcontroller from Texas Instruments is chosen as the microcontroller. Built around a 16-bit CPU, the MSP430 is designed for low cost, and specifically, low power consumption. The TI CC2420 RF transceiver is chosen for the wireless communication design. CC2420 is a true single-chip 2.4 GHz IEEE 802.15.4-compliant RF transceiver designed for low-power and low-voltage wireless applications which has been reported in many WSN node designs.

**Figure 6.**
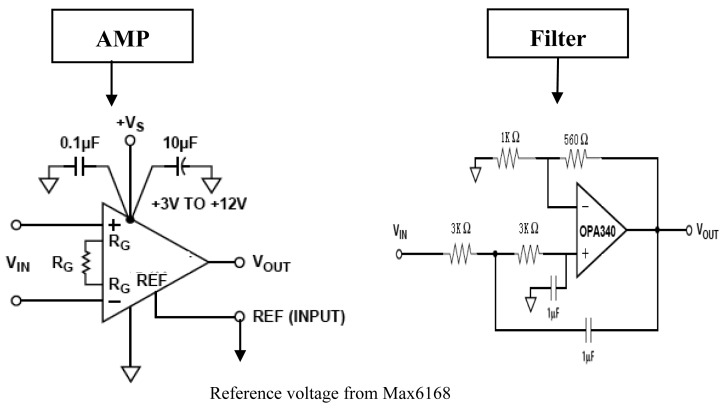
Circuit design of the amplifier and filter.

Figure 6 shows the detailed design of the amplifier and filter circuits. An LP2985 fixed-output voltage regulator is used to provide the bridge voltage. Since the sensitivity of the strain gauge is usually low, an instrumentation amplifier AD623 is adopted to amplify the bridge circuit output. As a low-power zero-drift instrumentation amplifier, the AD623 can offer excellent accuracy for sensor nodes. The WSN strain node is designed to measure strains in a range from −3000 με to 3000 με which is the ordinary strain measuring range requested in engineering applicationd. The Ref of the AD623 is designed to be connected with the output of a MAX6168 which provides a reference voltage to the amplifier in the AD623 as shown in Figure 6. The low-pass filter is designed using OPA340 chip to eliminate the high frequency noise. OPA340 is a single-supply operational amplifier from TI Company. It offers excellent dynamic response with low current consumption.

**Figure 7.**
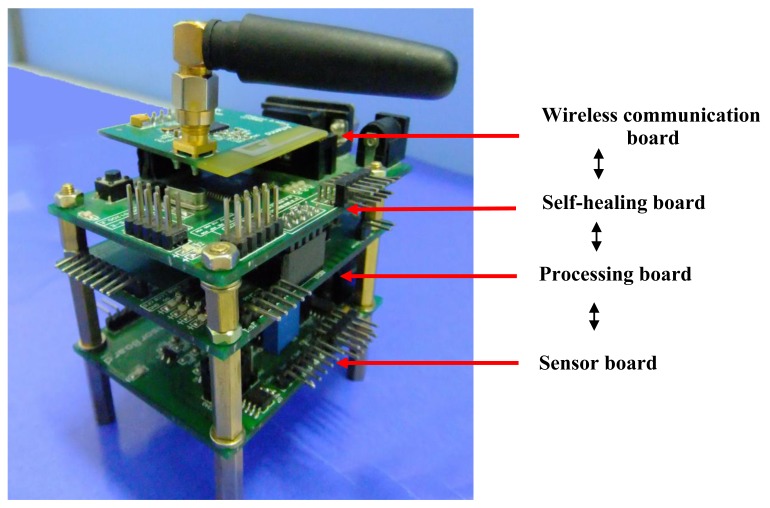
Redundancy-based self-healing WSN strain node developed.

Figure 7 shows the developed redundancy-based self-healing WSN strain node. To implement the self-healing ability, the main modules are not connected with each other directly as usual. Instead, they are connected with the FPAA chip. In the initial state, the FPAA is configured to connect I1P with O2P, I1N with 02N, I3P with O4P. In this case, the bridge circuit, the amplifier module 1 and the filter module 1 are connected with each other and the output of the filter chip OPA340 of filter module 1 is connected with ADC0. The node software reads the data from ADC0 in the normal state. When a fault happens, the microprocessor detects it and the FPAA is reconfigured dynamically to cut the connections to the failed component and replace it with another redundant module. In this paper, the amplifier module 2 and the filter module 2 are redundant modules. To evaluate whether Failure 1 happens, the output of the amplifier module 1 is also set to be connected with the ADC2 of the microprocessor.

In this research, AnadigmDesigner2 software is adopted to reconfigure the AN231E04 chip, which can quickly and easily construct complex analog circuits by selecting, placing and wiring together building block sub-circuits. The analog circuit configuration can be downloaded to the FPAA.

### 4.2. Failure Diagnosis and Self-Healing Process

**Figure 8.**
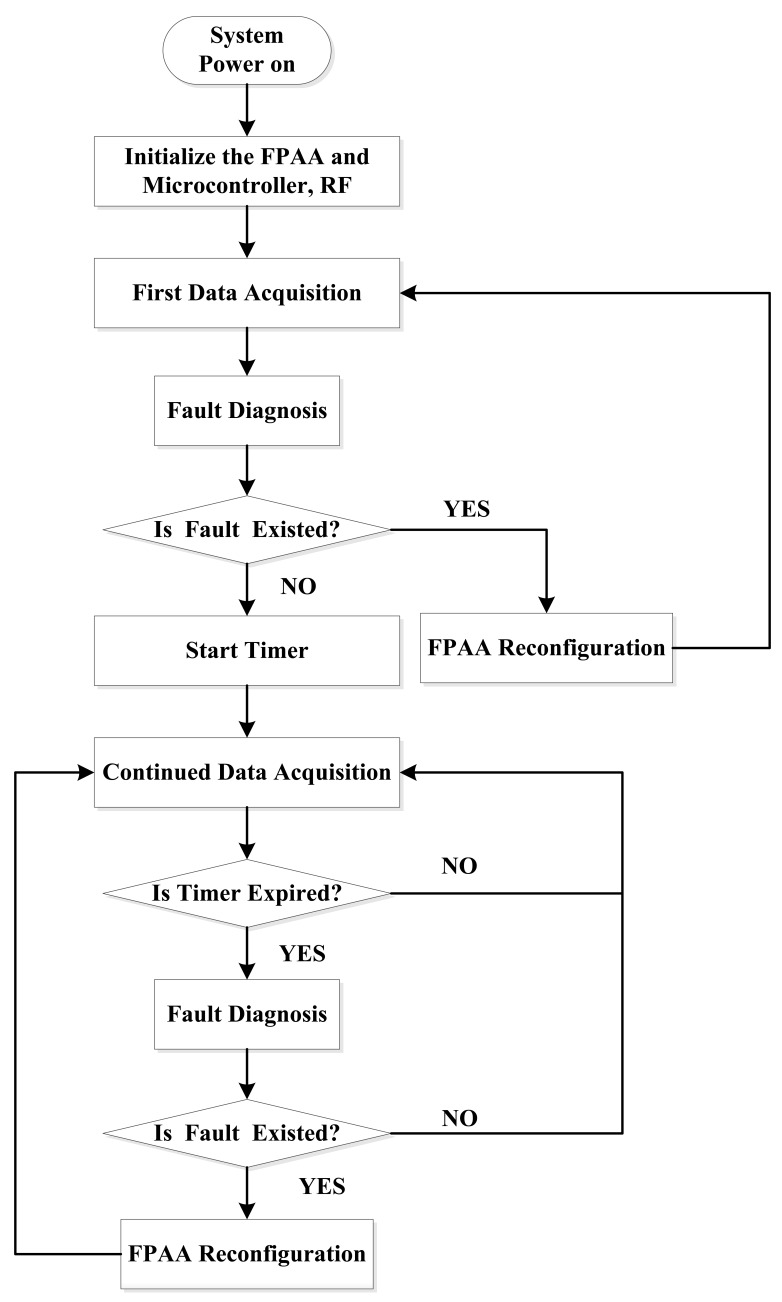
Software in MSP430 on the self-healing node.

Figure 8 shows the software flow chart of the self-healing node. When power is on, the node performs a fault check first and then it returns to its normal working state. During the service, the node performs the fault diagnosis periodically. A timer in the microprocessor is used to set the interval which is decided by the application request. Once a fault is found, the reconfiguration process will be triggered.

**Figure 9.**
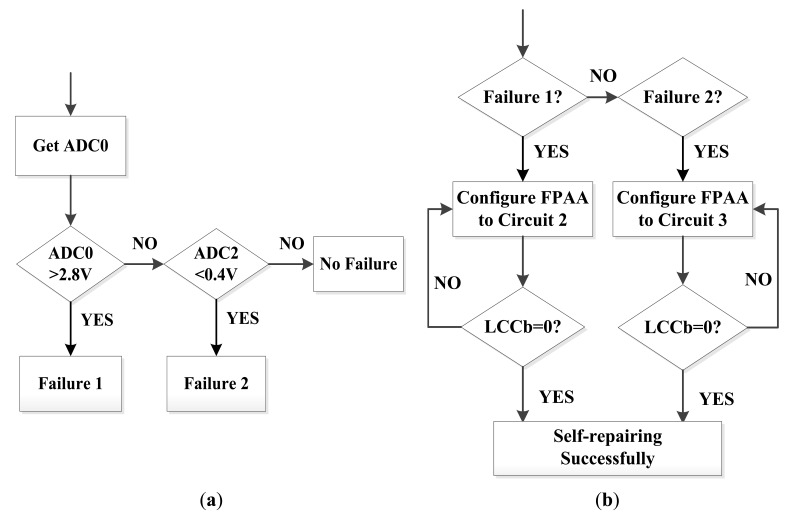
Failure diagnosis and self-healing process: (**a**) Fault diagnosis process (**b**) Reconfiguration process.

Figure 9 shows the flow chart of the fault diagnosis and reconfiguration process. In this design, the strain measuring range is ±3,000 με. This range is designed to correspond to 0.8 V–2.5 V output of the sensor board by adding an input bias voltage to the amplifier AD623. When the amplifier saturates, it outputs a voltage of 2.83 V in the situation when the voltage supplied to the amplifier is 3.3 V. When the filter circuit has connection failure, its output voltage is 0 V. Both above outputs are abnormal outputs and can be distinguished by the microprocessor software. Since the outputs of the AD623 and the filter circuit are connected with different A/D inputs of MSP430, the software distinguishes different module failures by reading different A/D converter outputs. If they are correct, the software returns to its normal sampling process. If not, the software distinguishes the fault modes and dynamically reconfigures the FPAA to have a new connection to recover the node.

In Figure 9, LCCb is the pin of the AN231E04. It indicates the successful finish of the dynamic configuration process when its value is 0. Table 2 shows the different circuit configurations during the whole process.

### 4.3. Design of the Whole FPAA-Based Self-Healing Strain Sensor Node

**Figure 10.**
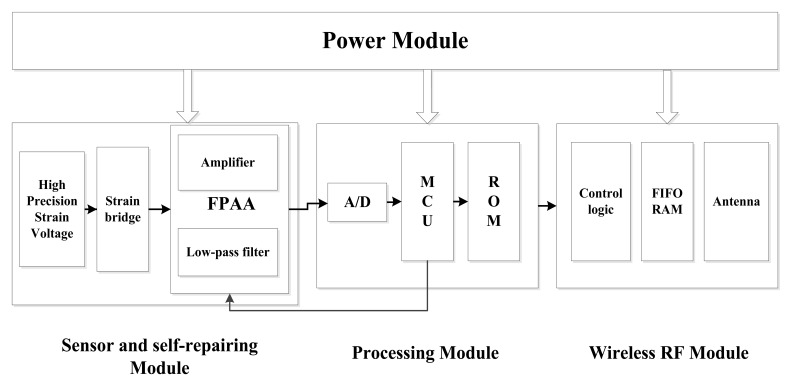
FPAA-based self-healing WSN strain node hardware structure.

In this session, the WSN strain sensor node is still adopted as illustration. The schematic structure of the self-healing WSN strain sensor node using the FPAA-based self-healing paradigm is shown in Figure 10. Different from the design in Section 4.2, the amplifier and filter circuits here are realized by modules in the AN231E04 FPAA chip. There are no additional amplifier and filter circuits needed.

**Figure 11.**
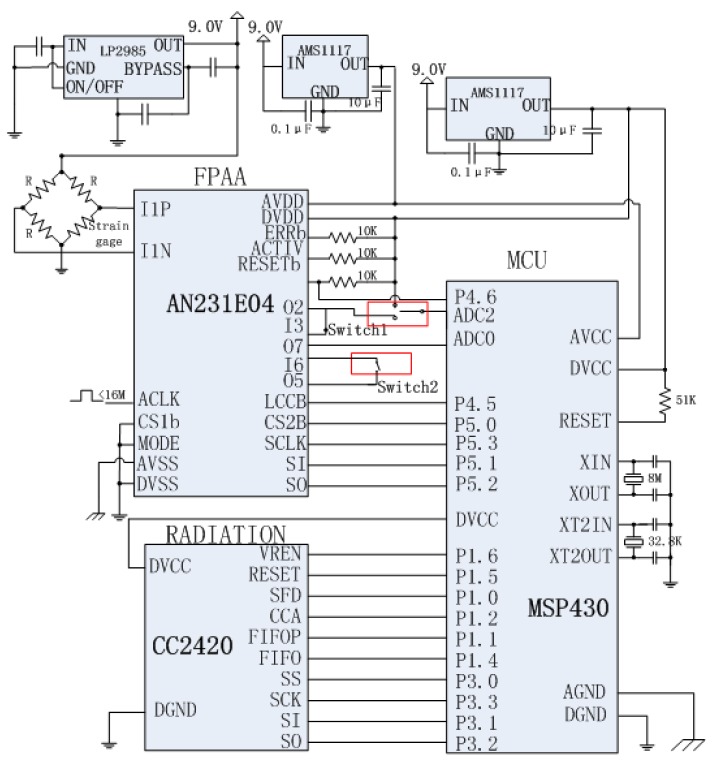
Circuit design of the FPAA-based self-healing node.

Figure 11 is the detailed design of the FPAA-based self-healing sensor node. Two switches are also adopted here to make the failures. Switch 1 is used to cut the connection of AMP_1 to the microprocessor and connects the 3.3 V to the ADC2 of the microprocessor to simulate the amplifier saturation failure. Switch 2 is used to cut the connection from Filter_1 to ADC0 of the microprocessor to produce the filter connection failure.

The software working process, the failure diagnosis and self-healing process of the node is the same as shown in Figures 8 and 9. Table 3 gives the circuit configurations under different states of this node.

**Figure 12.**
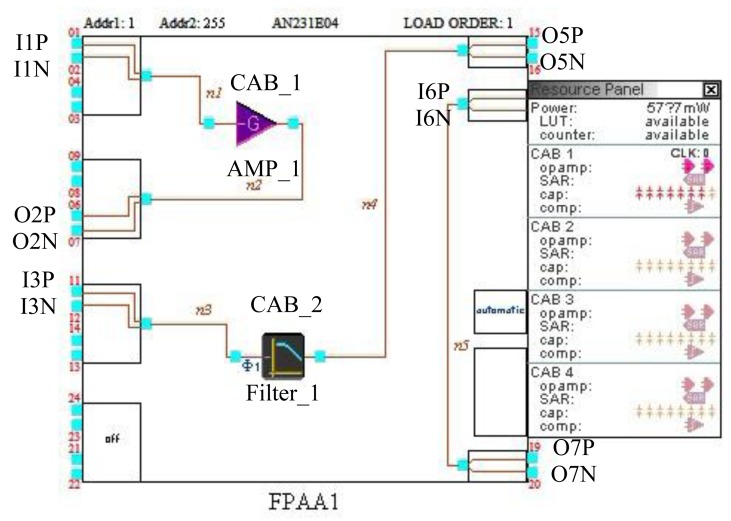
Circuit configuration of the FPAA in the normal state.

Figure 12 shows the internal circuits configured in the FPAA when the node is implemented and works in the normal state. In CAB1, an amplifier named as AMP_1 is configured and a low pass filter in CAB2 is configured labeled as Filter_1. In a normal working state, the output of Filter_1 is the O5 pin of the FPAA. In the circuit, it is connected with Switch 2 and Switch 2 is connected with the O6 pin. By connecting O6 with O7 in the FPAA, the output of Filter_1 can be connected with the ADC0 input of the MSP430.

When the amplifier saturation failure is detected, the software configures the internal circuits of the AN231E04 to Circuit 2. In this case, another amplifier labeled as AMP_2 is configured in CAB3 in the AN231E04 to replace the faulty amplifier. When a filter connection failure is detected, the software configures the internal circuits of the AN231E04 to Circuits 3. Another filter labeled as Filter_2 is realized in CAB4 to replace the faulty filter. The output of Filter_2 is configured directly to be O7.

**Figure 13.**
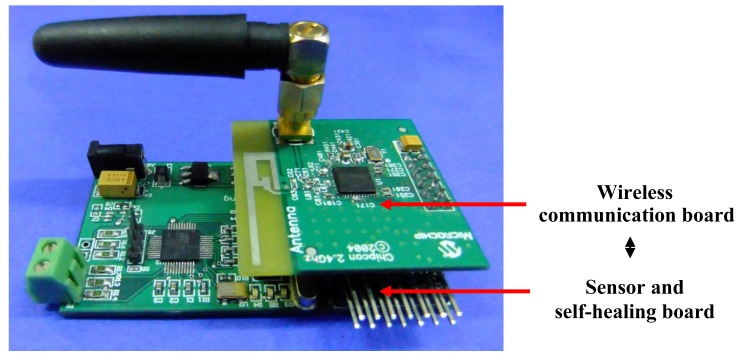
The developed FPAA-based self-healing WSN strain node.

Figure 13 shows the FPAA-based self-healing sensor node developed. Two boards are designed, namely a wireless RF board and sensor and self-healing board. The sensor and self-healing board integrates the bridge circuit, voltage regulator, the FPAA circuit and the microprocessor part.

## 5. Experimental Research

Two kinds of experiments are performed in this section to evaluate the developed self-healing WSN nodes. The first one is the node self-healing ability validation experiment. In this experiment, circuit failures are made intentionally to check the self-healing ability of the designed nodes. Another experiment is the energy consumption experiment. The energy consumption of the designed two nodes is tested and discussed.

### 5.1. Self-Healing Ability Demonstration Experiment

**Figure 14.**
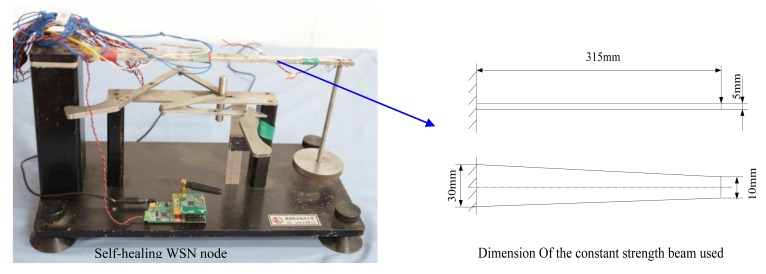
Constant strength beam-based experimental system.

A demonstration experiment is performed to evaluate the self-healing function of the developed nodes. A constant strength beam based experimental system is established, shown in Figure 14.

In Figure 14, one end of the constant strength beam is fixed. A load caused by a weight is applied on the other end. A strain gauge is arranged on the surface of the beam. It is connected to the bridge circuit of the self-healing nodes. Since this is a constant strength beam, strain caused on the surface of the beam along the axis direction is the same at any point. The strain gauge is arranged in the middle of the beam along the axis direction. A computer with a sink node is used as base station to receive data from the developed self-repairing nodes. A Telosb node is adopted here as the sink node.

Demonstration software is developed based on Labview software to control the sensing process, transfer data from the nodes to the base station and show the data received on the computer screen in real time.

To demonstrate the whole working process of the developed WSN nodes including normal working state and the self-healing state, an experimental process is designed as below:

- Normal working process: in this process, the nodes work normally to sample strain data and send data to the base station. The output voltage corresponding to the strain should be shown on the software interface. In the process, totally three kinds of weights are planned to be applied on the beam.
- Amplifier saturation and self-healing process: in this stage, the designed Switch 1 is switched to intentionally saturate the amplifier. Under this situation, the nodes output faulty data corresponding to the saturated voltage. Once the failure happens, the nodes diagnose it and start the self-healing process. After that, the nodes should return to their normal working state automatically.
- Filter connection failure and self-healing process: in this stage, the designed Switch 2 is switched to intentionally make the connection between the filter and the ADC fail. Similarly, the nodes output wrong data. After detection by the microprocessor, the self-healing process is supposed to be triggered and then the nodes should return to the normal state automatically.
- Normal working process: after the two self-healing process, the nodes work normally.

**Figure 15.**
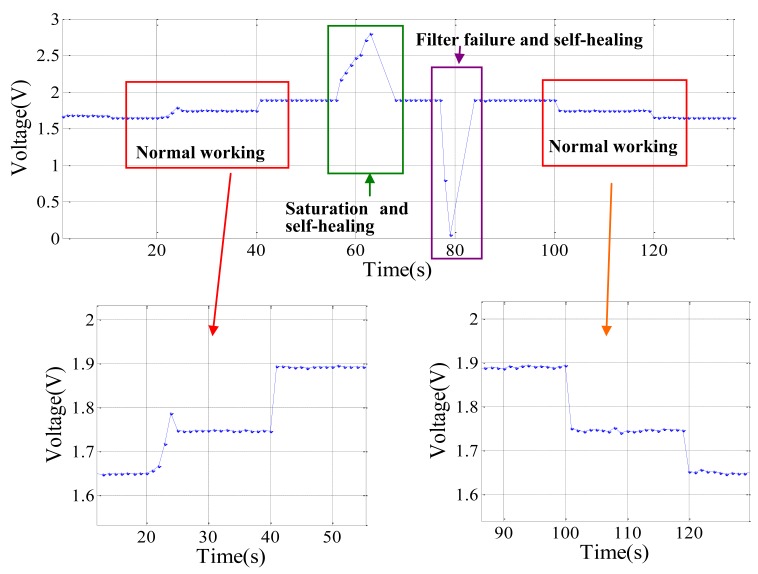
Output from the redundancy-based self-healing node during experiment.

During the experiment, the data sampling rate is set to 1 Hz. From the data sent from the node to the base station, the working process of the sensor nodes is demonstrated and each working stage can be distinguished clearly on the software interface. Figure 15 shows the data received by the base station which is output by the redundancy-based self-healing node.

At the first stage, the node works normally. When different weights are applied on one end of the constant strength beam, the node consequently monitors different strains. In this experiment, three weight grades 0, 1 kg and 2.5 kg, are applied, respectively, corresponding to 0, 342 με and 855 με strain caused on the beam and 1.65 V, 1.75 V and 1.89 V outputs from the sensor node. From the data shown in Figure 15, these data can be found easily. At the second stage, the amplifier saturation failure is intentionally cuased. In this case, the output of the node begins to increase and reaches a saturated voltage of 2.85 V. The software in the node diagnoses this failure and starts the self-healing process to replace the faulty amplifier with a new one. From the output of the node, it can be noticed that the output of the node gradually decreases to its normal state. In the third stage, Switch 2 is used to cut the connection of the filter to the AD converter. Under this situation, the output of the node first decreases to 0 V. After self-healing the node, the output increases to the normal state. In the fourth state, weights are applied again. The correct output of the node proves that it has been totally recovered.

**Figure 16.**
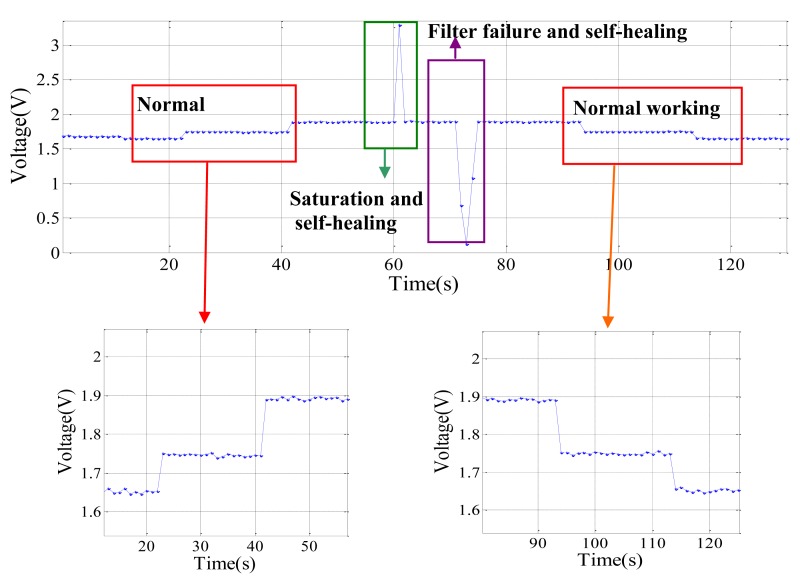
Output of the FPAA-based self-healing node during experiment.

Figure 16 shows the data received by the base station which is output by the FPAA-based self-healing node. The process is similar to Figure 15. A small difference is that when the node suffers an amplifier saturation failure, the node output is 3.3 V. This is because the amplifier saturation failure of this node is simulated by connecting a 3.3 V voltage to the ADC by Switch 1. Four working stages of the node can also be distinguished clearly on the data received by the base station on the software interface. It can be noticed that the recovery speed of the FPAA-based self-healing design is quicker than the redundancy-based self-healing design. The main reason is that the circuit switching speed between internal modules in FPAA is faster than the circuit switching speed of redundant modules in redundancy-based nodes. These experiments show that the nodes developed can self-diagnose failures and recover to their normal working state automatically.

### 5.2. Power Consumption Evaluation

Power consumption of the developed self-healing nodes is also evaluated by experiments. Regarding the redundancy-based self-healing node, it surely consumes more energy than the FPAA self-healing node because it uses redundancy modules and it costs more energy when more redundancy modules are adopted.

**Figure 17.**
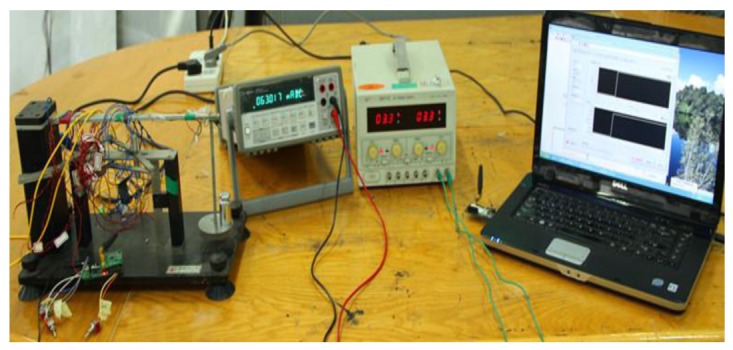
Experiment system to test power consumed.

The experimental setup is shown in Figure 17. Figure 17 shows the experiment system setup. A YX1715A voltage power supply is used to supply a fixed 3.3 V voltage to the self-healing nodes. A 34401A digital multi-meter from Agilent is adopted to measure the current the node consumed from the constant voltage power supplier. A computer-based base station is adopted here to receive the data sent by the node.

During the experiment, the power consumed is measured in a typical working state: the nodes sample strain at a sampling frequency of 10 Hz. After each sampling, the nodes send the data wirelessly to the base station. A Telosb node which has the same MSP430 microprocessor and the CC2420 chip is also tested in the experiment using the same setup. Table 4 shows the measurement results. By checking the manual of the FPAA chip AN231E04, it can be learned that the standard current of the AN2331E04 consumed at working state is approximate 42 mA. The increasing part of the current consumption of the FPAA based self-healing node comparing to ordinary Telosb node is just because the adoption of the FPAA chips. The standard currents of the OPA340, AD623 and MAX6168 chip are 2.3 mA, 1.5 mA, 5 mA, respectively. The redundancy-based self-healing node has two OPA340 chips and two AD623 chips, therefore, it consumes more energy compared to the FPAA-based self-healing node.

Though the power consumption of the designed self-healing nodes have increased, the nodes can still work for a reasonable period. For example, if the FPAA-based node is supplied power by two 1,700 mAh AA batteries and the node samples strain for 10 s at an interval of 1 h every day, it still can work for about 9 months. For those applications where the battery can be recharged, replaced or can be equipped with energy harvesting devices, such as structural health monitoring, industrial process monitoring, healthcare applications and home automation, the power consumption of the self-healing nodes can be totally acceptable. Besides, with the rapid development of reconfigurable hardware, low power consumption reconfigurable hardware may also emerge in the near future.

## 6. Conclusions

For many applications of WSNs, such as structural health monitoring, industrial process monitoring, healthcare applications, home automation, *etc.*, the batteries of the wireless sensor nodes can be replaced at a certain interval or recharged. In these situations, energy consumption is no longer a very critical problem, but if these nodes may have to work in harsh environments or serve for a long term, the robustness of the WSN node is very important to its successful applications. To improve the robustness of the WSN nodes and reduce their maintenance costs, a new concept of self-healing node based on reconfigurable hardware is proposed in this paper. Two kinds of reconfigurable hardware-based self-healing WSN node realization paradigms are presented and the nodes are developed. Their sizes are 9 × 6.5 × 5.5 cm and 7 × 4.5 × 2 cm, respectively, which are just ordinary WSN nodes' size. Demonstration experiments are performed and show that the nodes developed can self-diagnose the failures and recover to normal working state automatically. Further research can be done to improve the proposed methods including:

- The hardware of the self-repairing wireless sensor nodes can be improved. In this paper, only two typical analog module failures are used to demonstrate the self-repairing methods. In future designs, both FPAAs and FPGAs can be adopted to self-heal both analog and digital circuits.
- Since only two analog circuits are chosen in this paper to demonstrate the self-healing concept, the failure diagnostic method in the software is somewhat simple. For more modules' self-healing situation, the failure diagnostic software should be improved to distinguish different module failures reliably and efficiently.
- The self-repairing performance parameters during the design of the self-healing WSN nodes are not researched in detail yet. The speed of the self-repair during the self-repairing process can be addressed in depth in future work.

## Figures and Tables

**Table 1.** Failure modes.

**Failure Index**	**Failure Modes**	**Circuit Status**
1	Amplifier failure	Amplifier saturation
2	Low-pass filter failure	Filter connection failure

**Table 2.** Circuit configurations of redundancy-based self-healing WSN node.

**Circuit 1**	**Circuit 2**	**Circuit 3**
I1P to O2P	I1P to O5P	I1P to O2P
I1N to O2N	I1N to O5N	I1N to O2N
IP3 to O4P	I6P to O4P	IP3 to O7P

**Table 3.** Circuit configurations of FPAA-based self-healing WSN node.

**Circuit 1**	**Circuit 2**	**Circuit 3**
I1 P/N to AMP_1	I1 P/N to AMP_2	I1 P/N to AMP_1
AMP_1 to O2P	AMP_2 to O2P	AMP_1 to O2P
I3 P/N to Filter_1	I3 P/N to Filter_1	I3 P/N to Filter_2
Filter_1 to O5P	Filter_1 to O5P	Filter_2 to O7P

**Table 4.** Power consumption test results.

**Node Type**	**Test Results**	

	**Working Voltage (V)**	**Working State (mA)**
Telosb node (Mps430+CC2420)	3.3	22.1
FPAA based Self-healing node (Mps430+CC2420+AN231E04)	3.3	62.7
Redundancy-based self-healing node (Mps430+CC2420+AN231E04+OPA340+AD623+MAX6168)	3.3	75.5
